# The effect of harvesting the anterior half of the peroneus longus tendon on foot morphology and gait

**DOI:** 10.1186/s13018-023-04429-6

**Published:** 2024-01-16

**Authors:** Zhi Zhao, Li Tang, Jing Chen, Xinwen Bai, Yu Chen, Liqi Ng, Yu Zhou, Yu Deng

**Affiliations:** 1 Department of Sport Medicine, Chongqing Orthopedic Hospital of Traditional Chinese Medicine, Chongqing, 400012 China; 2 Postdoctoral Research Workstation, Chongqing Orthopedic Hospital of Traditional Chinese Medicine, Chongqing, 400012 China; 3 Chongqing Rongzhi Biotechnology Company Limited, Chongqing, 400012 China; 4 Institute of Orthopaedic and Musculoskeletal Science, University College London, Royal National Orthopaedic Hospital, Stanmore, London, HA7 4LP UK

**Keywords:** Peroneus longus tendon, Anterior cruciate ligament reconstruction, Ankle, Gait

## Abstract

**Background and objectives:**

In anterior cruciate ligament reconstruction, the strength of the graft was found to be unsatisfactory usually the anterior half of the peroneus longus tendon was taken for supplementation, but the effect on foot and ankle function and gait in the donor area is unclear. This study aims to explore the changes in the ankle and gait after using the harvested anterior half of the peroneus longus tendon as a reconstruction graft for the anterior cruciate ligament.

**Methods:**

A total of 20 patients, 6 males and 14 females, aged 18 to 44 years, with unilateral anterior cruciate ligament injuries, underwent reconstruction using the harvested anterior half of the peroneus longus tendon as a graft between June 2021 and December 2021. The part on which the anterior half of the peroneus longus tendon was harvested was considered the experimental group, while the contralateral foot was the control group. At the 6-month follow-up, the Lysholm knee score, AOFAS ankle score, and gait-related data (foot length, arch index, arch volume, arch volume index, and gait cycle parameters: percentage of time in each gait phase, step frequency, step length, foot strike angle, and push-off angle) were assessed using a 3D foot scanner and wearable sensors for both groups.

**Results:**

All 20 patients completed the six-month follow-up. There were no statistically significant differences between the experimental and control groups regarding knee scores, ankle scores, foot length, arch index, arch volume, arch volume index, step frequency, and step length (*P* > 0.05). However, there were statistically significant differences between the experimental and control groups in terms of the gait cycle parameters, including the percentage of time in the stance, mid-stance, and push-off phases, as well as foot strike angle and push-off angle (*P* < 0.05).

**Conclusion:**

Through our study of the surgical experimental group we have shown that harvesting the anterior half of the peroneus longus tendon does not affect foot morphology and gait parameters; however, it does impact the gait cycle.

## Introduction

Anterior cruciate ligament (ACL) injuries are one of the most common knee injuries, leading to anterior and rotational instability of the knee, which further increases the chances of causing meniscus tears and cartilage injuries, among other things [1]. Anterior cruciate ligament reconstruction is an internationally recognised treatment modality for restoring knee stability. There are various types of grafts for ligament reconstruction, such as autologous tendons, allograft tendons, or artificial ligaments [2, 3]. As far as autologous tendons are concerned, the popliteus tendon and peroneus longus tendon are chosen more often [4, 5]. However, there is a lack of clarity regarding the effect on foot and ankle function and gait after autologous peroneus longus tendon excision and most of the current scholars believe that this effect is minor or non-existent, but they are relatively limited in the methods of assessment they use, such as functional scores, X-rays, and so on [6, 7]. In particular, in the study by Marín et al. [8], it was noted that whether there is an effect on the foot and ankle after peroneal longissimus tendon excision needs to be further confirmed with stronger evidence using more reliable assessment tools.

Gait analysis is a state-of-the-art modality for assessing foot and ankle function. It is widely recognised that it provides information about the subject's level of function during movement compared to traditional static assessment on imaging and subjective scoring systems, and that it is more reflective of the patient's motor function status and has been widely used in all aspects of clinical research [9–12]. The current gold standard technology used for gait analysis is optoelectronic systems, but these are usually found in large laboratories because they are expensive and take up a lot of space [13, 14]. The most cost-effective method currently available is the use of an entire Inertial Measurement Unit (IMU) [15] consisting of a gyroscope, accelerometer, and magnetometer or parts thereof to detect motion data, which has utility as well as simplicity, leading wearable devices to become more popular, and by which can measure more foot characteristics, and miniaturisation and mobile sensor technology are satisfactory for detection results [15, 16]. In this study, we hypothesised that the anterior half of the peroneus longus tendon may affect foot shape, gait parameters, or gait cycle after reconstruction of the anterior cruciate ligament as a supplemental graft, so we used a 3D scanner and wearable gait sensors to detect the changes in foot shape and gait after the anterior half of the peroneus longus tendon was cut.

## Materials and methods

### Patient selection

Informed consent from the patient was approved and signed by the hospital ethics committee; between June 2021 and December 2021, 20 consecutive patients underwent ACL injury reconstruction surgery with parallel excision of the anterior half of the peroneus longus tendon as a ligament reconstruction graft. Inclusion criteria included patients with a confirmed diagnosis of ACL injury with more than 50% or more rupture and requiring an anterior half cut of the peroneus longus tendon as a ligament reconstruction graft (popliteal tendon braided diameter ≤ 7 mm). The foot after cutting the anterior half of the peroneus longus tendon was set up as the experimental group and the healthy foot as the control group. The exclusion criteria included: (1) poor functional recovery 6 months after surgery; (2) the presence of flat feet, high arched feet, horseshoe feet, bunions, plantar fasciitis and other foot diseases; (3) the combination of other neuromuscular diseases or other diseases involving the nerves (e.g. connective tissue disease, diabetes mellitus, spinal or pelvic diseases); (4) patients with a history of previous lower limb surgery; (5) subjects who refused to sign the informed consent form.

### Method of cutting the anterior half of the peroneus longus tendon

**Fig. 1:**
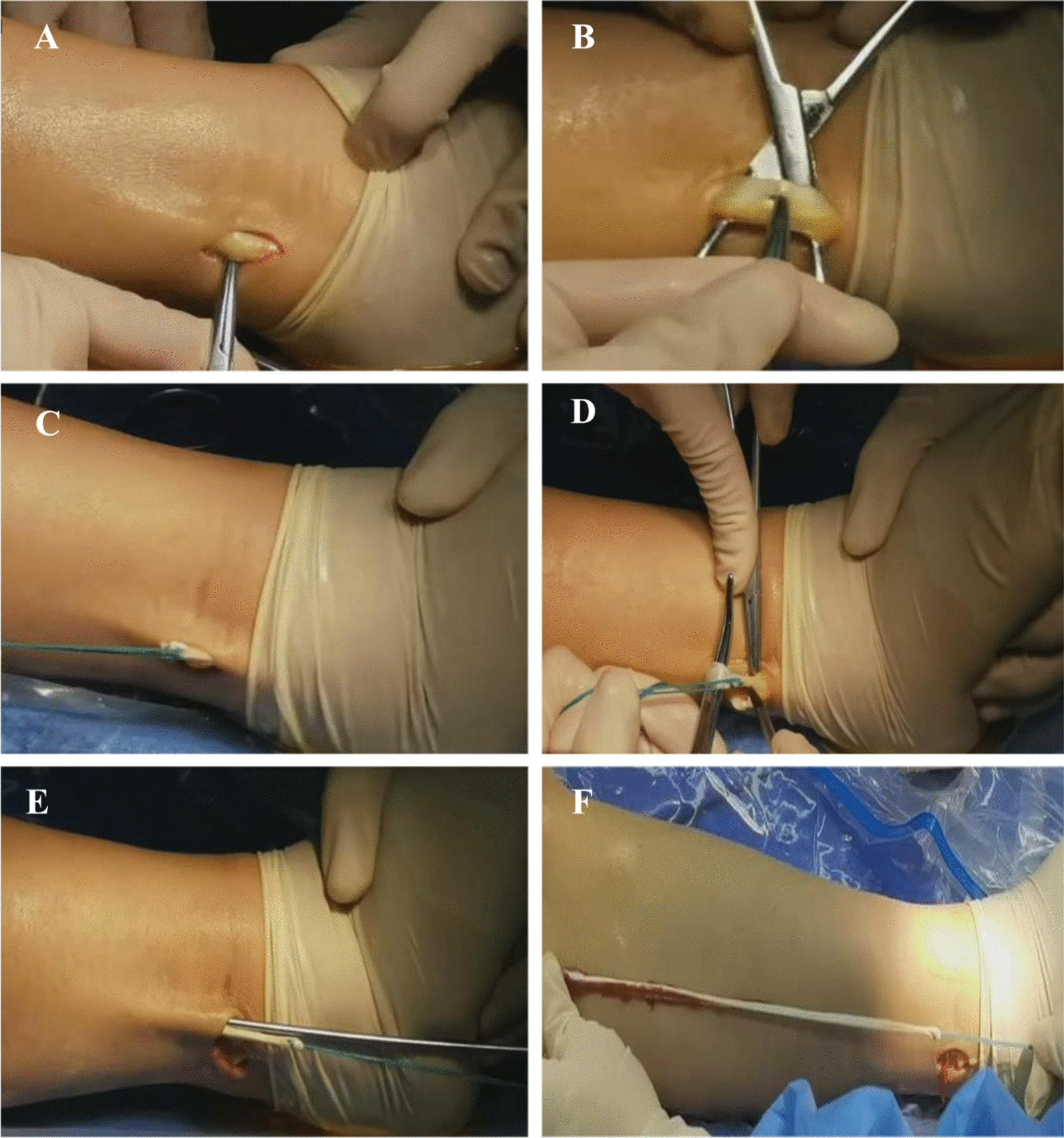
Steps for cutting the anterior half of the peroneus longus tendon. **A** An incision was made above the posterior aspect of the lateral ankle tip to reveal the peroneus longus tendon; **B** the anterior half of the tendon was picked out with hemostatic forceps; **C** the selected portion of the tendon was tied with No. 2 Ethibond sutures; **D** the ankle was dorsally extended and turned outward, and the distal end of the tendon was sufficiently revealed to dissect the anterior half of the tendon; **E** the distal end of the tendon was implanted in the tendon retriever, and the tendon was stripped along the peroneal alignment in the direction of the head of the peroneal bone; **F** the anterior half of the tendon was removed in its entire length

The surgery was performed by joint surgeons. An incision is made 2 cm above the tip of the lateral ankle to expose the peroneus longus tendon, the anterior portion of the tendon is picked out with hemostatic forceps, and the selected portion of the tendon is tied with a No. 2 Ethibond suture (Johnson & Johnson, USA), the ankle is dorsally extended and turned outward, and the distal end of the tendon is fully exposed and the anterior portion of the tendon is severed. The distal end of the tendon is implanted into a tendon retriever, and the tendon is stripped along the fibular alignment in the direction of the fibular head and then placed onto the tendon table to strip away the muscular tissues. If the tendon is difficult to cut (usually because the proximal end of the tendon is too thick for the tendon stripper to push), a 1-cm incision is made 5 cm below the fibular head, and a perforated guide needle is inserted into the initial incision against the surface of the deep fascia, and a polyethylene suture with a braided tendon is inserted into the loop of the perforated guide needle and pulled out against the surface of the proximal incision (Figs. 1).

### Postoperative rehabilitation

For 1–2 weeks postoperatively, the patient locks the knee in full extension with an adjustable brace at rest, and partial to full weight bearing is performed with crutches as tolerated (if meniscal suture surgery was performed at the same time, full weight bearing is delayed until the 4th postoperative week). On the other hand, ankle pump training, quadriceps, hamstring isometric contraction training, and patellar internal thrust training were performed; passive knee flexion mobility training was required, requiring passive flexion activities in full range, increasing knee flexion mobility by 10–15° per day, and finally reaching ≥ 120° of knee flexion. It was also combined with physical therapy including ice and infrared therapy. Three to 4 weeks after surgery, the patient used an adjustable brace to lock the knee joint in the fully extended position during rest and braced double crutches for full weight bearing. Straight leg raising training, hamstring isometric contraction training, heel lift training; knee passive flexion mobility training, requiring further increase in knee flexion angle to 130°; proprioceptive training, such as support gait training, pedalling fixed clip bicycle and so on. At 5–8 weeks postoperatively, the patient locks the knee in full extension with the same adjustable brace at rest, fully weight bearing within the tolerable range, and gradually abandons the crutches if the patient begins to develop a pain-free and normal gait pattern. Passive knee flexion mobility training further increases the angle of knee flexion to reach the healthy side level; weight-bearing straight leg raise training, semi-squatting training, popliteal strength training and other functional exercises; proprioceptive training, such as balance board training, gait training with a brace, pedalling fixed clip bicycle and so on. At 9–12 weeks after surgery, the brace can be removed, but be careful to avoid knee hyperextension while walking. Active knee flexion mobility training, semi-squatting training, active knee extension training in sitting position, and hamstring muscle strength training, etc.; strengthen the proprioceptive training, such as balance board training, and pedal clip bicycle; strengthen the patient's dexterity training, such as side stepping steps. From 13 weeks to 6 months after surgery, patients were instructed to perform quadriceps strength training and hamstring strength training; proprioceptive training was the same as the previous stage and flexibility training was carried out, such as forward jogging at a constant speed [17].

### Postoperative functional assessment

**Fig. 2:**
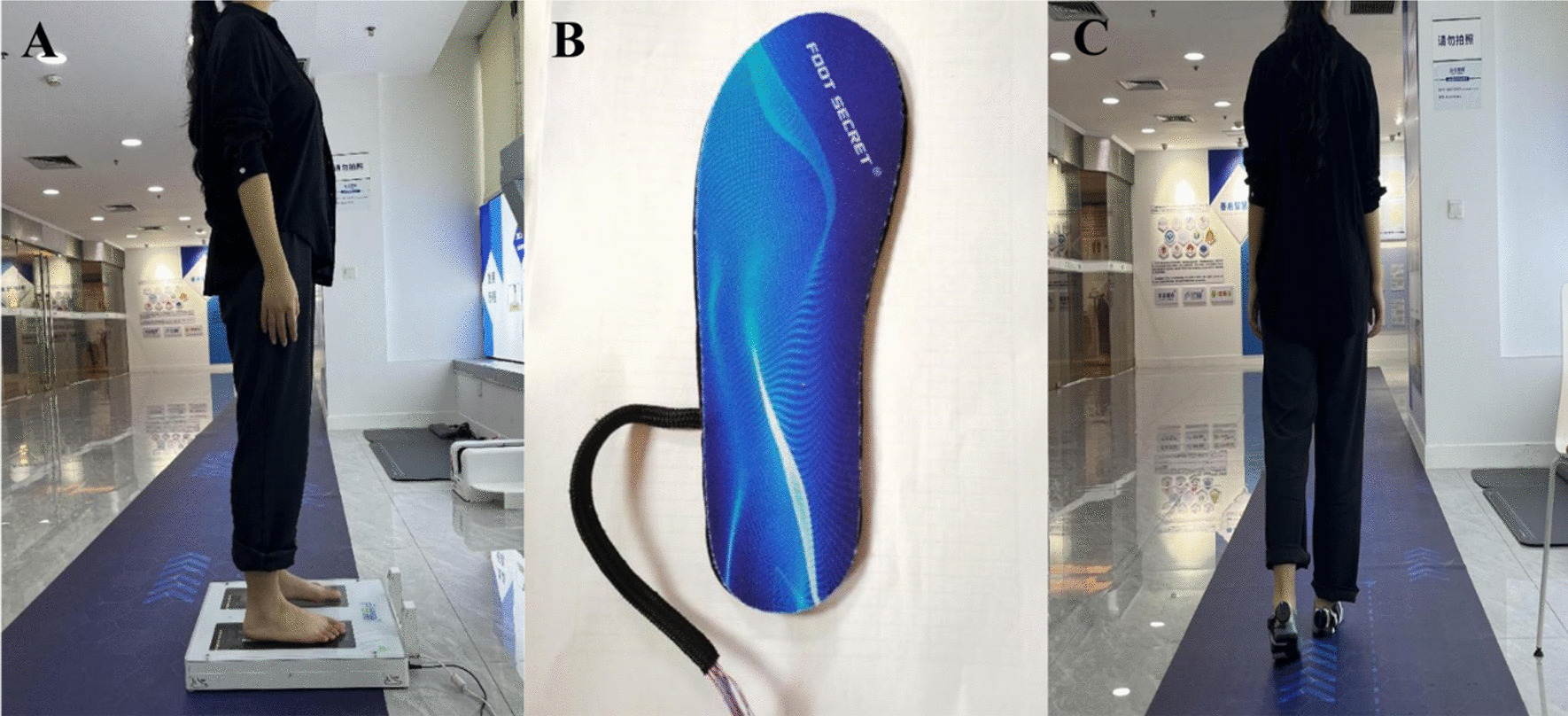
3D foot scanner and wearable gait sensor. **A** 3D model of the foot captured with a 3D scanner; **B** wearable gait sensor insoles; **C** subjects were allowed to wear gait sensors and walk in a natural state on a trail

**Fig. 3:**
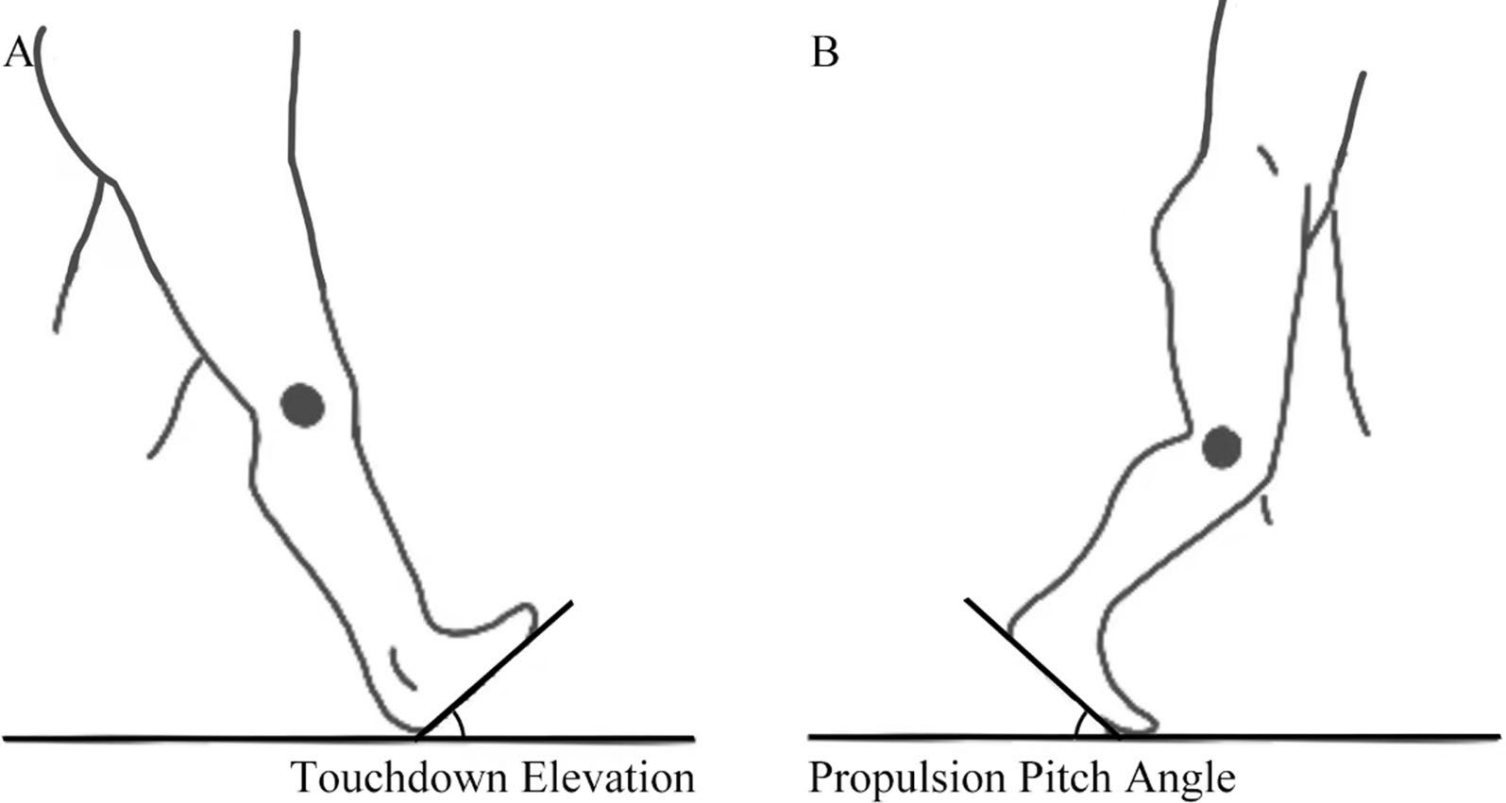
Schematic diagram of touchdown elevation and propulsion pitch angle. **A** the sagittal plane angle between the foot and the ground at the beginning of the touchdown phase is the touchdown elevation angle; **B** The sagittal plane angle between the foot and the ground at the end of the propulsion phase is the propulsion pitch angle

Follow-up was performed 6 months after surgery, and the Lysholm score was used to assess the functional recovery of the knee after ACL reconstruction; the American Orthopaedic Foot & Ankle Society (AOFAS) [18, 19] score was used to assess the functional recovery of the foot and ankle after the anterior half of the peroneus longus tendon was cut; the 3D model of the foot was captured with a 3D scanner, and the arch index and arch volume were collected and the arch volume index was calculated; the subjects were allowed to wear gait sensors (a sensor accurately measured). The gait sensor (a fixation device for accurate sensor measurement, Shanqi, China) was allowed to be worn by the subjects, and they walked on the trail in a natural walking state for two minutes, and the sensor automatically generated the gait parameters of the experimental group and the control group to the computer in the cloud, then data were downloaded, and gait parametric (stride frequency, stride amplitude) data were collected in relation with the gait cycle [20, 21] (the time percentage of the various stages of the gait cycle, the elevation angle of touching the ground, and the propulsion angle of pitching the ground) (Figs. 2, 3).

### Statistical analysis

Based on our clinical experience, SPSS 21.0 statistical software was used for data analysis. Normally distributed measures were expressed as mean ± standard deviation ($\overline{\mathrm{x}}$ ± s); for continuous variables, paired-samples t test or rank-sum test (when normality or Chi-square was not satisfied) was used. *P* < 0.05 differences were statistically significant.

## Result

### General information about the patient

Between June 2021 and December 2021, a total of 20 patients with unilateral ACL injuries with anterior half of peroneus longus tendon excision as ligament reconstruction grafts were admitted to our department, including 6 males and 14 females, with ages ranging from 18 to 44 years old, mean age of 29.05 ± 8.33 years old, mean height of 163.55 ± 7.13 cm, mean body weight of 57.65 ± 10.35 kg, and the average body mass index was 21.41 ± 2.57 (Table 1).

**Table 1:** General information about the patient

Patient characteristics	*N*	Average value	(Statistics) standard deviation	Minimum value	Maximum values
Age	20	29.05	8.33	18.00	44.00
High(cm)	20	163.55	7.13	151.00	175.00
Weight(kg)	20	57.65	10.35	37.00	76.00
BMI (kg/m^2^)	20	21.41	2.57	15.20	25.26

### Functional evaluation of the knee joint six months after surgery

There were no complications such as incision infection, deep vein thrombosis of the lower limb, and internal fixation detachment in all 20 patients. The Lysholm function score of the knee joint of the operated side was (95.90 ± 2.13), and that of the healthy side was (96.50 ± 1.85), and the difference was not statistically significant (*P* > 0.05). Foot and ankle joint function assessment six months after surgery, no foot and ankle complications occurred in 20 patients, and the AOFAS scores were (98.05 ± 1.73) on the operated side and (98.30 ± 1.66) on the healthy side, with no statistically significant difference (*P* > 0.05) (Table 2).

**Table 2:** Comparison of bilateral knee Lysholm function scores and foot and ankle AOFAS function scores of the subjects

Subject	Operative side		Healthy side		Test value	*P* value
	Average value	(Statistics) standard deviation	Average value	(Statistics) standard deviation		
Lysholm score	95.90	2.13	96.50	1.85	− 1.21	0.240
AOFAS score	98.05	1.73	98.30	1.66	− 1.31	0.204

### Six months postoperative 3D scanner measurements

The foot shape of the operated side and the healthy side was measured with a 3D scanner in 20 patients, in which the foot lengths were (234.29 ± 9.15) mm and (234.09 ± 9.08) mm for non-weight bearing, (237.38 ± 9.53) mm and (237.38 ± 9.04) mm for weight bearing, and (0.22 ± 0.06) for non-weight bearing, and (0.22 ± 0.06) for the foot arch index, respectively (0.24 ± 0.04), the arch index at weight bearing was (0.25 ± 0.04), (0.26 ± 0.04), the arch volume at non-weight bearing was (21,704 ± 6880) mm^3^, (22,585 ± 6931) mm^3^, the arch volume at weight bearing was (17,096 ± 5560) mm^3^, (17,579 ± 5387) mm^3^, and the arch volume index was (0.21 ± 0.12), (0.21 ± 0.11), respectively. There was no statistically significant difference in bilateral comparison of the above data (*P* > 0.05) (Table 3).

**Table 3:** Comparison of subjects’ bilateral 3D plantar scanner measurement metrics

Subject	Operative side		Healthy side		Test value	*P* value
	Average value	(Statistics) Standard deviation	Average value	(Statistics) Standard deviation		
Length of foot (mm)	234.29	9.15	234.09	9.08	− 5	0.852
Weight bearing-foot Length (mm)	237.38	9.53	237.38	9.04	0.5	0.993
Arch index	0.22	0.06	0.24	0.04	18.5	0.471
Weight bearing-arch index	0.25	0.04	0.26	0.04	27	0.249
Arch volume (mm^3^)	21,704	6880	22,585	6931	17	0.546
Weight bearing-arch volume (mm^3^)	17,096	5560	17,579	5387	0.85	0.406
Arch volume index (AVI)	0.21	0.12	0.21	0.11	0.13	0.895

### Gait sensor measurements six months postoperatively

Twenty patients wore gait sensors to measure the gait-related indexes of the surgical side and the healthy side, respectively, in which the time percentage of the Stance phase was (58.34 ± 4.90)%, (59.68 ± 4.34)%, the time percentage of the swing phase was (41.66 ± 4.90)%, (40.32 ± 4.34)%, and the frequency of steps was (80.31 ± 20.09) steps/min, (83.12 ± 10.45) steps/min, and stride length was (108.25 ± 13.88)cm, (109.84 ± 13.07) cm, respectively. The differences of the above data were not statistically significant when compared bilaterally (*P* > 0.05). On the other hand, the percentage of time in mid-stance was (30.29 ± 4.21)%, (29.47 ± 3.33)%, the percentage of time in touchdown phase was (10.95 ± 2.34)%, (11.87 ± 1.66)%, the percentage of time in advancement phase was (17.10 ± 2.75)%, (18.34 ± 2.79)%, and the angle of elevation of touchdown during touchdown was (11.06 ± 4.84)° and (13.02 ± 4.94)°, and the advancing pitch angle of the advancing phase was (44.60 ± 7.70)° and (49.80 ± 5.38)°, respectively. The difference between the above data in bilateral comparison was statistically significant (*P* < 0.05) (Table 4).

**Table 4:** Comparison of subjects’ bilateral wearable gait sensor measurement metrics

Subject	Operative side		Healthy side		Test value	*P* value
	Average value	(Statistics) standard deviation	Average value	(Statistics) standard deviation		
Touchdown phase (%)	10.95	2.34	11.87	1.66	3.47	0.003
Mid-stance phase (%)	30.29	4.21	29.47	3.33	− 58	0.030
Propulsion phase (%)	17.10	2.75	18.34	2.79	84	< .001
Stance phase (%)	58.34	4.90	59.68	4.34	47	0.083
Swing phase (%)	41.66	4.90	40.32	4.34	− 47	0.083
Frequency of steps	80.31	20.09	83.12	10.45	7.5	0.738
Stride length	108.25	13.88	109.84	13.07	0.61	0.549
Touchdown period—touchdown elevation angle (°)	11.06	4.84	13.02	4.94	2.55	0.019
End of propulsive phase—propulsive pitch angle (°)	44.60	7.70	49.80	5.38	4.07	< .001

## Discussion

While the focus of most research attention has been limited to the subjective means of scoring tools, the present study identified changes in more objective quantitative metrics utilizing a 3D scanner and wearable gait sensors, i.e. differences in the gait cycle between some of the data sets and the athlete's foot after cutting the anterior half of the peroneus longus tendon. Previous studies have shown that the peroneus longus tendon is one of the optional complementary grafts for ACL reconstruction surgery of the knee when the popliteal tendon is not strong enough and has better results in terms of function and graft survival and is currently available in both total and anterior half cuts [22, 23]. According to Nazem.K, there is no significant effect on foot and ankle function after total resection of the peroneal longissimus tendon, but this finding lacks support from long-term follow-up [24]. In the study by Zhao et al. [25], they concluded that the ACL could be reconstructed by cutting the anterior half of the peroneus longus tendon in combination with the popliteal tendon to minimize the impact on the ankle. The foot and ankle function was evaluated using the foot and ankle AOFAS score and the Foot and Ankle Disability Index (FADI) score, and it was concluded that the anterior half of the peroneus longus tendon did not affect the function of the foot and ankle after resection. Similarly, in our study, the postoperative AOFAS score was evaluated for foot and ankle function and there was no significant difference.

However, both of these scores are more subjective assessment tools and lack objective quantitative indicators of the foot and ankle [8, 26]. For example, the scoring mentions the gait abnormality grading, with 8 points for none or slight, 4 points for significant, and 0 points for very significant, and the ankle-hindfoot stability grading, with 8 points for stable, and 0 points for significantly unstable. It is not very easy for the assessor to delineate the specific boundaries between each of the grades mentioned above, and there is a wide range of scores between grades, as well as a lack of clarity in the definitions of gait abnormality and ankle-hindfoot stability, which can only be scored by subjective perception by patients with a lack of expertise. The peroneus longus tendon has a role in stabilising the medial column of the foot and preventing excessive pronation of the foot during walking, reacting to sudden pronation of the foot, so the peroneus longus tendon may play an important role in stabilising the arch of the foot. Although the peroneus longus tendon is only one of the structures that stabilise the arch of the foot, it is the only tendon that maintains the arch of the foot by passing through the plantar aspect of the foot. When the joints of the foot become tightly packed together, the foot is better supported and forces are transmitted, and it is the peroneus longus tendon that participates in the maintenance of the arch of the foot by increasing the tension and dependence between the joints of the mid-foot [27, 28]. In our study, we found that after cutting the anterior portion of the peroneus longus tendon, there was no significant difference in foot length, arch index, and arch volume between the weight-bearing and non-weight-bearing sides of the affected limb compared to the healthy side, and we hypothesised that this might be related to the location of the tendon cut. The plantar terminus of the peroneus longus tendon is located on the plantar side of the medial cuneiform bone and the lateral side of the inferior aspect of the first metatarsal, and the anterior portion of the peroneus longus tendon was dissected at the level of the lateral ankle tip without affecting the plantar portion of the peroneus longus tendon. Therefore, tension could still be transmitted from the origin of the tendon to the plantar aspect of the foot through the residual posterior portion of the tendon, and the maintenance of the arch of the foot by the peroneus longus tendon was not affected [7]. At the same time, our opinion is in agreement with He et al. [22] that the peroneus longus tendon is severed at the proximal end of the outer ankle, the plantar portion of the tendon is left intact and the distal portion will be sutured to the peroneus brevis tendon, so there is no effect on the ankle after tendon excision.

Gait analysis is currently one of the most common ways to dynamically detect foot and ankle motion data, and wearable gait sensors have the advantage of being easier, more accurate, and more comprehensive [29–31]. We used a gait analysis device wearable gait sensor to collect dynamic data from patients. In terms of dynamic function, the peroneus longus tendon has a role in plantar flexion and external rotation of the ankle joint, and the gait cycle may be affected by tendon excision [6]. The gait cycle is the process from the landing of one side of the foot to the landing of that side of the foot again, which is divided into the stance phase and the swing phase [32–34]. The stance phase is divided into the touchdown phase, the mid-stance phase, and the propulsion phase, of which the stance phase is the phase in which the foot and ankle play a role [35, 36]. In our study, we also did not find any significant difference in the proportion of time spent in the swing phase between the operative side and the healthy side. During the touchdown phase, the heel just touches the ground with dorsiflexion of the ankle joint and simultaneous valgus due to the synergistic action of the peroneus longus tendon, which if insufficiently valgus will result in a reduction of the dorsiflexion moment [6]. In our study, we found that the average touchdown elevation angle of the affected foot during the gait cycle was reduced by approximately 2° compared to the healthy foot with a statistically significant difference (*P* < 0.05), which may be due to the weakening of the foot's eversion force after the anterior half of the peroneus longus tendon is cut, which is also following the study findings of Shao and Angthong et al. [26, 37]. During the transition from the mid-stance to the propulsive phase, the talonavicular joints remain rotated anteriorly for most of the time, and when the ground reaction forces diminish in the latter part of the mid-stance, the talonavicular joints begin to rotate posteriorly, thus performing a phase transition: i.e. from an active adaptive function required in the touchdown phase to a strong leverage function in the propulsive phase [38]. Contraction of the peroneus longus tendon causes dorsiflexion and valgus of the dice bone, thus locking the lateral aspect of the foot, maintaining the stable state of the lateral longitudinal arch of the foot, and guaranteeing effective power transmission [39]. We detected a statistically significant difference by gait cycle timeshare test, which showed that the mean touchdown period timeshare of the affected side was 0.92% shorter than that of the healthy side, while the mid-stance timeshare was 0.82% longer. We hypothesise that the foot takes longer to adapt during the transition from the mid-stance to the propulsive phase and less time to complete the propulsive manoeuvre, both of which may be related to the weakened muscle force contraction of the peroneus longus tendon after the anterior half of the tendon is cut [40]. After the onset of the propulsion phase, the peroneus longus tendon completes the propulsion by plantarflexing the first metatarsophalangeal joint and the lateral column of the pronator teres [41]. This is because the first metatarsal is shorter than the other metatarsals and must be plantarflexed to maintain contact with the ground. In plantarflexion, the transverse axis of the first metatarsophalangeal joint moves backward and upward, so that the lesser phalanx can reach the optimal dorsiflexion angle without hindrance [38]. In our study, we found that the average propulsive pitch angle on the operated side decreased by 4.2° (*P* < 0.001), the propulsive period time percentage decreased by 1.24% (*P* < 0.001) compared with that on the healthy side, and we hypothesised that this may also be related to the weakening of the foot's metatarsal flexion force after the peroneus longus tendon was cut in half. As the metatarsal flexion angle of the metatarsal bone decreased, the dorsal extension angle of the lesser toes also became smaller, and the vertical stress on the ground during foot propulsion also decreased accordingly, which affected walking efficiency [42, 43]. On the other hand, in our study, it was shown that there was no significant difference in gait parameters such as mean step frequency and stride length between the healthy side and the affected side, which is the same as most of the scholars have concluded [43, 44]. At the same time, the results of this gait analysis may have included changes in gait from the knee surgery itself, and although most studies have shown no difference in postoperative Lysholm scores, few comparisons of quantitative metrics have been made to rule out the effect of ligament surgery on gait. The study by Kaur et al. [45] reported that they compared the lower extremity mechanics of the affected limb and the healthy limb after ACL reconstruction and found no significant differences in kinematics at 5 years postoperatively, but there was not yet a complete recovery of the lower extremity abduction and adduction moments. Finally, there are limitations in this study, such as the small number of subjects and the short follow-up period. Our next step is to need a large amount of data and long-term follow-up to further evaluate the effect of cutting the anterior half of the peroneus longus tendon on foot and ankle function.

## Conclusion

In summary, the effect of cutting the anterior half of the peroneus longus tendon on the arch index, arch volume, and arch volume index was not significant, and the gait parameters of stride frequency and stride length did not produce significant changes. However, the patient's postoperative gait cycle showed significant changes in the touchdown elevation angle, the propulsion pitch angle, and the percentage of time spent in the touchdown, mid-stance, and propulsion phases. This difference in perception may be small or even absent during normal short walks but may be more pronounced during prolonged walks or certain sports (e.g. soccer, basketball, etc.). Therefore, we recommend choosing whether or not to use the anterior half of the peroneus longus tendon as a supplement during ACL reconstruction based on the patient's varying athletic demands, which can be used by those with low athletic demands and should be handled with caution by sports enthusiasts or athletes. This idea can be further confirmed in future experiments by increasing the testing time and walking speed of each subject.
